# Proceedings of the 2014 MidSouth Computational Biology and Bioinformatics Society (MCBIOS) Conference

**DOI:** 10.1186/1471-2105-15-S11-I1

**Published:** 2014-10-21

**Authors:** Jonathan D Wren, Mikhail G Dozmorov, Dennis Burian, Andy Perkins, Chaoyang Zhang, Peter Hoyt, Rakesh Kaundal

**Affiliations:** 1 Arthritis and Clinical Immunology Research Program, Oklahoma Medical Research Foundation; 825 N.E. 13th Street, Oklahoma City, OK 73104-5005, USA; 2 Biochemistry and Molecular Biology Dept, University of Oklahoma Health Sciences Center, USA; 3 Stephenson Cancer Center, University of Oklahoma Health Sciences Center, USA; 4 Department of Geriatric Medicine, University of Oklahoma Health Sciences Center, USA; 5 Civil Aerospace Medical Institute, Federal Aviation Administration, Oklahoma City, OK 73169, USA; 6 Department of Computer Science and Engineering, Mississippi State University, Mississippi State, MS 39762, USA; 7 School of Computing, University of Southern Mississippi, Hattiesburg, MS 39406, USA; 8 Department of Biochemistry & Molecular Biology, Oklahoma State University, Stillwater, OK 74078, USA; 9 Bioinformatics Facility, Institute for Integrative Genome Biology, Department of Botany & Plant Sciences, University of California, Riverside, CA 92521, USA; 10 Virginia Commonwealth University, Richmond Academy of Medicine, Department of Biostatistics, USA

## Introduction

The MidSouth Computational Biology and Bioinformatics Society (MCBIOS 2014) held its eleventh annual conference at the Wes Watkins Center at Oklahoma State University, Stillwater on March 7-8, 2014. The theme was "*From Genome to Phenome: Connecting the Dots*". Conference Chair this year was Rakesh Kaundal, who is also one of the MCBIOS board members, and conference committee members were Ulrich K. Melcher and Doris Kupfer. The current president is Andy Perkins and Cesar Compadre was elected as President-Elect for 2015-16. There were 154 registrants and a total of 125 abstracts submitted (50 oral and 75 poster presentations).

Keynote speakers were Owen White from The University of Maryland School of Medicine, whose talk was titled "The Human Microbiome Project: Large-Scale Data Management and Analysis", and Jody Hey from Temple University "Designing Genealogy Samplers for Population Genetics". Dr. William Slikker, Director of the Food and Drug Administration's, National Center for Toxicological Research, concluded with a talk on the past ten years of MCBIOS and a perspective on its future.

Participants also had the opportunity to attend a workshop on next-generation sequencing (NGS), hosted by Peter Hoyt of OSU and Dr. Graham Wiley of the Oklahoma Medical Research Foundation. The workshop had a keynote by Dr. Joshua Orvis of The University of Maryland School of Medicine and Johns Hopkins University on genome annotation.

The winners of conference awards were:

Best Paper Award: Zongliang Yue, Ping Wan, Hui Huang, Zhan Xie and Jake Y. Chen for "SLDR: A Method to Identify New Gene Regulatory Relationship Candidates" [1]

Best Paper Runner-up: Nam S Vo and Vinhthuy Phan for "Exploiting dependencies of pairwise-comparison outcomes to predict patterns of gene response" [2]

Best Oral Presentations (Post-Doctoral fellows):

Michael A. Bauer, University of Arkansas for Medical Sciences

Erich A. Peterson, University of Arkansas for Medical Sciences

Best Oral Presentations (students):

Karl Walker, Arkansas State University, 1^st ^place Shraddha Thakkar, University of Arkansas for Medical Sciences, 2^nd ^place

Mihir Jaiswal, University of Arkansas at Little Rock, 3^rd ^place

Best Poster (Computation):

Stephen Reichley, Mississippi State University, 1^st ^place

Kushal Bohra, Texas A&M University at Commerce, 2^nd ^place

Austin McCullough, John Brown University, 3^rd ^place

Best Poster (Biology):

Shraddha Thakkar, University of Arkansas for Medical Sciences, 1^st ^place

Sunetra Das, University of Oklahoma, 2^nd ^place (tie)

Garima Saxena, University of North Texas, 2^nd ^place (tie)

Kangmei Zhao, University of Oklahoma, 2^nd ^place (tie)

## Selecting papers for the MCBIOS XI Proceedings

All papers were anonymously peer-reviewed by at least 2 reviewers and quantitatively evaluated on the basis of three criteria: Novelty, Impact and Clarity, enabling papers to be ranked. Editors who were also co-authors of submitted papers were not permitted to handle their own papers editorially. This year, 22 papers were submitted, and 16 papers were deemed acceptable by reviewers, giving an acceptance rate of 73%, higher than last year's 59%. Papers generally fell into four categories:

## Networks and pathways

Zongliang Yue, *et al*. [1] developed a new computational technique called Step-Level Differential Response (SLDR) to identify genetic regulatory relationships. This technique takes advantages of functional genomics data for the same species under different perturbation conditions, therefore complementary to current popular computational techniques. It can particularly identify "rare" activation/inhibition relationship events that can be difficult to find in experimental results. SLDR is computationally efficient with o(N2) complexity and may be applied to the mining of functional genomics big data for future network biology and network medicine applications.

The manuscript by Toby and colleagues [3] reports some interesting observations regarding the possible role of the SigB regulon in the divergence of members of the *Bacillus cereus *group based on the protein-coding content of 25 completed whole genomes of *B. cereus *group isolates. The authors used cluster analysis of orthologous proteins to reconstruct the clade structure of the *B. cereus *group, and found that the resulting structure follows the pattern of what genes belong to the SigB regulon or not. Their observation suggests the hypothesis that horizontal gene transfer, gene duplication/divergence and deletion dictate the underlying coding capacity in these genomes, and is likely a common pattern in prokaryotic evolution.

Peterson *et al*. developed a workflow based on whole exome sequencing to discover clonal lineages in tumor samples. Here, they utilize the availability of samples from a single multiple myeloma patient from initial presentation and two subsequent relapses to arrive at key single nucleotide and insertion/deletion events in Key Genes that presumably gave rise to the initial presentation and the relapses. What is unique is the visualization tool, CloneViz, which allows for visualization of these events by chromosome and applies Gaussian kernel density plots for easy comparison between samples. The benefit is a better understanding of the evolution of a cancer[4].

## Genomics & transcriptomics

In light of a shrinking NIH budget, researchers can stretch funds further if they can decrease the number of samples used for gene expression profiling. The paper by Vo and Phan [2] addresses the issue of small sample size in microarray experiments that contain measurements of multiple responses. While they developed their method for microarray studies, it can well be scaled to gene expression profiling using RNA sequencing technology. The latter requires complex data processing, made easy by the paper of Peng *et al*. Their software, SeqAssist [5], processes raw FASTQ files and extracts useful biological data. Zhang et. al. used two other state-of-the-art pipelines to analyze whole genomes, by sequencing 35 Korean individuals [6]. Not only did they identify genomic variants specific for the Korean population, but also analyzed functions of genes and disease susceptibility associated with these SNPs.

Michael A. Bauer, *et al*. describe custom software tools for the integration and analysis of data from various molecular profiling methods. This software allows the integration of microarray, RNA-Seq, and whole exome sequencing data, and was applied here to sample from a patient with multiple myeloma for illustrative purposes[7].

## Proteomics

The manuscript by Jaiswal and co-authors [8] presents a new algorithm for analyzing cross-linking mass spectrometry data, XLPM, and its implementation. The results of applying the new software to model systems are shown. Central to this approach is the B-y ion filter which the authors have used to analyze NIST spectral library. The authors claim the XLPM selection filter leads to increased analysis speed and higher confidence non-specific cross-link data.

The manuscript by Zhang and Zhao [9] performed some quality measurements on mass-spectrometry metabolomics data. They conclude that their proposed zigzag index is better than several metrics including the MCQ index in evaluating the quality of extracted ion chromatographs (EICs). This work is important due to a need for effective metrics of quality evaluation of EICs to filter out low-quality data.

Millions of dollars are spent annually to better understand how pathogens infect their hosts and to identify potential targets for therapeutics. Protein-Protein Interaction (PPI) is an important mechanism playing a crucial role in host-pathogen interactions and pathogenicity. Although there are several bioinformatics methods developed to predict PPIs at the intra-species level, there are very few studies at the inter-species level. As a case study on Arabidopsis-*Pseudomonas syringae *interaction system, Sitanshu S. Sahu *et al *[10] developed various domain and interolog based approaches to predict genome-scale PPI network. This is the first report of deciphering an inter-species interactome in any plant-microbe system.

Jo and Cheng [11] discussed an important problem in proteome science i.e. protein fold recognition. A random forest based machine learning method is proposed to predict the fold of proteins and they demonstrated its efficiency by comparing their method with the existing approaches. The method should help in recognizing the correct structural fold for template-based protein structure modeling.

Laccases (E.C. 1.10.3.2) are multi-copper oxidases that have gained importance in many industries such as biofuels, pulp production, textile dye bleaching, bioremediation, and food production. Previous classification systems for laccase enzymes are based on multiple sequence alignments and they largely follow species taxonomy rather than substrate ranges, enzyme properties, or specific function. In the manuscript by Weirick *et al *[12], they developed a two-phase classification system; first using unsupervised learning approaches to identify various laccase subtypes based on sequence features and enzyme function, and then developed a supervised learning method for predicting/classifying new laccases from the unknowns. This tool will be a useful resource to the biotechnology community especially those working in the area of biofuels.

Huiwen Ng *et al*. [13] developed a competitive docking approach (CDA) for performing ligand-docking in Estrogen receptors. The CDA takes into account and compares the non-covalent interactions between a specific ligand and the two separate docking models based on the respective docking scores of the docked complex and, therefore, better reflects the receptor-ligand interaction. The CDA approach is extensible to other receptor targets both to screen for potential binders and to differentiate between agonists and antagonists, and is as applicable in drug discovery as for regulatory testing purposes.

## Miscellaneous

Thousands of bioinformatics programs have been published and put online, but studies have shown that many of these resources become inaccessible in a time-dependent manner [14]. Jason Hennessey *et al*. conduct the largest survey to date of the current availability of previously published URLs - over 27,000. They attempt to identify which ones are Scientific Data Analysis Resources (SDARs) and analyze factors associated with the probability they are still available. They find that SDAR production tends to be distributed widely among institutions (in contrast to publications in general, the bulk of which tend to come from a relatively small fraction of institutions), and that more authors per SDAR-producing paper tends to equate with a greater probability of future accessibility [15].

Weizhong Zhao *et al*. discuss methods of summarization of text datasets in the form of topic modeling and show its effectiveness on three separate biological datasets [16].

Stephen Grace *et al*. present Haystack, a web-based tool for metabolomics research. Haystack is designed to visualize, parse, filter, and extract significant features from Liquid Chromatography Mass Spectrometry (LCMS) datasets. They show it is effective when comparing proteomics data from plants grown under two different light conditions [17].

## Future meetings

MCBIOS XII will be held at The Statehouse Convention Center in Little Rock, Arkansas in 2015 from March 12-14, tentatively entitled "*Emerging Trends in Bioinformatics*". The 2014-2015 MCBIOS President is Chaoyang "Joe" Zhang from the University of Southern Mississippi. MCBIOS is a regional affiliate of the International Society for Computational Biology (http://www.ISCB.org). For information regarding MCBIOS and our future meetings, see http://www.MCBIOS.org.

## Competing interests

The authors declare that they have no competing interests.

## Declaration of funding

Funding for the publication of this editorial was authorized by and obtained from the Mid-South Computational Biology and Bioinformatics Society.

## Authors' contributions

All authors served as editors for these proceedings, with JDW serving as Senior Editor. All authors helped write this editorial.
